# Integrated Myofibrillar Protein Synthesis in Recovery From Unaccustomed and Accustomed Resistance Exercise With and Without Multi-ingredient Supplementation in Overweight Older Men

**DOI:** 10.3389/fnut.2019.00040

**Published:** 2019-04-11

**Authors:** Kirsten E. Bell, Matthew S. Brook, Tim Snijders, Dinesh Kumbhare, Gianni Parise, Ken Smith, Philip J. Atherton, Stuart M. Phillips

**Affiliations:** ^1^Department of Kinesiology, University of Waterloo, Waterloo, ON, Canada; ^2^School of Life Sciences, University of Nottingham, Nottingham, United Kingdom; ^3^Department of Human Biology, NUTRIM School of Nutrition and Translational Research in Metabolism, Maastricht University, Maastricht, Netherlands; ^4^Department of Medicine, University of Toronto, Toronto, ON, Canada; ^5^Exercise Metabolism Research Group, Department of Kinesiology, McMaster University, Hamilton, ON, Canada; ^6^School of Graduate Entry Medicine and Health, University of Nottingham, Derby, United Kingdom

**Keywords:** fractional synthesis rate, deuterated water, resistance exercise training, high-intensity interval training, whey protein, creatine, vitamin D, n-3 PUFA

## Abstract

**Background:** We previously showed that daily consumption of a multi-ingredient nutritional supplement increased lean mass in older men, but did not enhance lean tissue gains during a high-intensity interval training (HIIT) plus resistance exercise training (RET) program. Here, we aimed to determine whether these divergent observations aligned with the myofibrillar protein synthesis (MyoPS) response to acute unaccustomed and accustomed resistance exercise.

**Methods:** A sub-sample of our participants were randomly allocated (*n* = 15; age: 72 ± 7 years; BMI: 26.9 ± 3.1 kg/m^2^ [mean ± SD]) to ingest an experimental supplement (SUPP, *n* = 8: containing whey protein, creatine, vitamin D, and n-3 PUFA) or control beverage (CON, *n* = 7: 22 g maltodextrin) twice per day for 21 weeks. After 7 weeks of consuming the beverage alone (Phase 1: SUPP/CON only), subjects completed 12 weeks of RET (twice per week) + HIIT (once per week) (Phase 2: SUPP/CON + EX). Orally administered deuterated water was used to measure integrated rates of MyoPS over 48 h following a single session of resistance exercise pre- (unaccustomed) and post-training (accustomed).

**Results:** Following an acute bout of accustomed resistance exercise, 0–24 h MyoPS was 30% higher than rest in the SUPP group (effect size: 0.86); however, in the CON group, 0–24 h MyoPS was 0% higher than rest (effect size: 0.04). Nonetheless, no within or between group changes in MyoPS were statistically significant. When collapsed across group, rates of MyoPS in recovery from acute unaccustomed resistance exercise were positively correlated with training-induced gains in whole body lean mass (*r* = 0.63, *p* = 0.01).

**Conclusion:** There were no significant between-group differences in MyoPS pre- or post-training. Integrated rates of MyoPS post-acute exercise in the untrained state were positively correlated with training-induced gains in whole body lean mass. Our finding that supplementation did not alter 0–48 h MyoPS following 12 weeks of training suggests a possible adaptive response to longer-term increased protein intake and warrants further investigation. This study was registered at ClinicalTrials.gov.

**Clinical Trial Registration:** www.ClinicalTrials.gov, identifier: NCT02281331

## Introduction

Reduced muscularity, a component of sarcopenia (1), appears to be driven to a large extent by the relative resistance of older skeletal muscle to the anabolic effects of loading (i.e., resistance exercise) (2) and protein ingestion (3). Relatively large bolus doses (compared to younger persons) of at least 0.4–0.5 g/kg/meal of protein are required to optimally stimulate myofibrillar protein synthesis (MyoPS) following resistance exercise in older muscle (3). To combat sarcopenic muscle loss, recommendations often suggest combining daily protein supplementation with resistance exercise training (RET). Although this combined strategy has yet to be proven with protein alone (4), multi-nutrient supplementation combined with RET may be effective in preventing and treating sarcopenia (5). We (6) and others (7–9) have shown that RET plus aerobic exercise or high-intensity interval training (HIIT) combined with multi-ingredient supplementation supports lean mass and strength gains in various groups of older adults (overweight, sarcopenic, healthy). Furthermore, the regular practice of both exercise modalities along with multi-ingredient supplementation induces other physiological changes important for healthy aging, such as increased cardiovascular fitness, heightened insulin sensitivity, and reduced inflammation (6, 10).

We previously reported that 6 weeks of ingesting a whey protein-based multi-ingredient supplement increased both appendicular and trunk lean body mass [measured by dual-energy X-ray absorptiometry (DXA)] and strength in a group of older men (6). Yet the subsequent completion of 12 weeks of combined RET + HIIT did not further increase regional or whole body lean mass (6). Although DXA does not measure skeletal muscle directly, increases in lean tissue mass generally align with hypertrophy (11). As such, it is possible that augmented rates of MyoPS underpinned the initial gains in whole body lean mass; however, the specific response of MyoPS to several weeks of multi-ingredient supplementation (with and without exercise training) is unknown. Importantly, we previously reported a high degree of concordance between integrated MyoPS and hypertrophy (12).

The main objective of this study was to determine whether daily integrated rates of MyoPS in response to acute resistance exercise would be altered following regular consumption of a multi-ingredient nutritional supplement alone and in combination with multimodal exercise training. A secondary objective was to examine the association between integrated rates of MyoPS and indicators of muscle hypertrophy during exercise training. We hypothesized that supplementation would stimulate MyoPS to a greater extent than a control beverage independently but not when combined with multimodal exercise training. We further hypothesized that integrated rates of MyoPS would correlate positively with training-related changes in DXA measurements of lean mass and muscle fiber cross-sectional area (CSA).

## Methods

### Participants

The present study was a distinct sub-analysis of participants from our original trial (6), which was approved by the Hamilton Integrated Research Ethics Board and registered at ClinicalTrials.gov as NCT02281331. From the 49 participants in the original trial, we screened and recruited 15 healthy non-smoking men, all ≥65 years, each of whom gave their written and informed consent to participate. During a 75 g oral glucose tolerance test, fasting plasma glucose was normal (<5.6 mM; *n* = 7 and *n* = 4 in the supplemented and control groups, respectively) or elevated (5.6–6.0 mM; *n* = 1 and *n* = 3 in the supplemented and control groups, respectively). Two-hour plasma glucose (2hPG) concentrations were normal (<7.8 mM) in all participants (13). No participants were diabetic or prediabetic. Resting blood pressure was <140/90 mmHg in all subjects. Exclusion criteria included regular use of non-steroidal anti-inflammatory drugs, use of simvastatin, and injury or chronic illness that would prevent safe participation in the study. Additionally, subjects were excluded if they regularly consumed any of the following dietary supplements: whey protein, creatine, calcium, vitamin D, or n-3 PUFA.

### Study Overview

**Figure 1:**
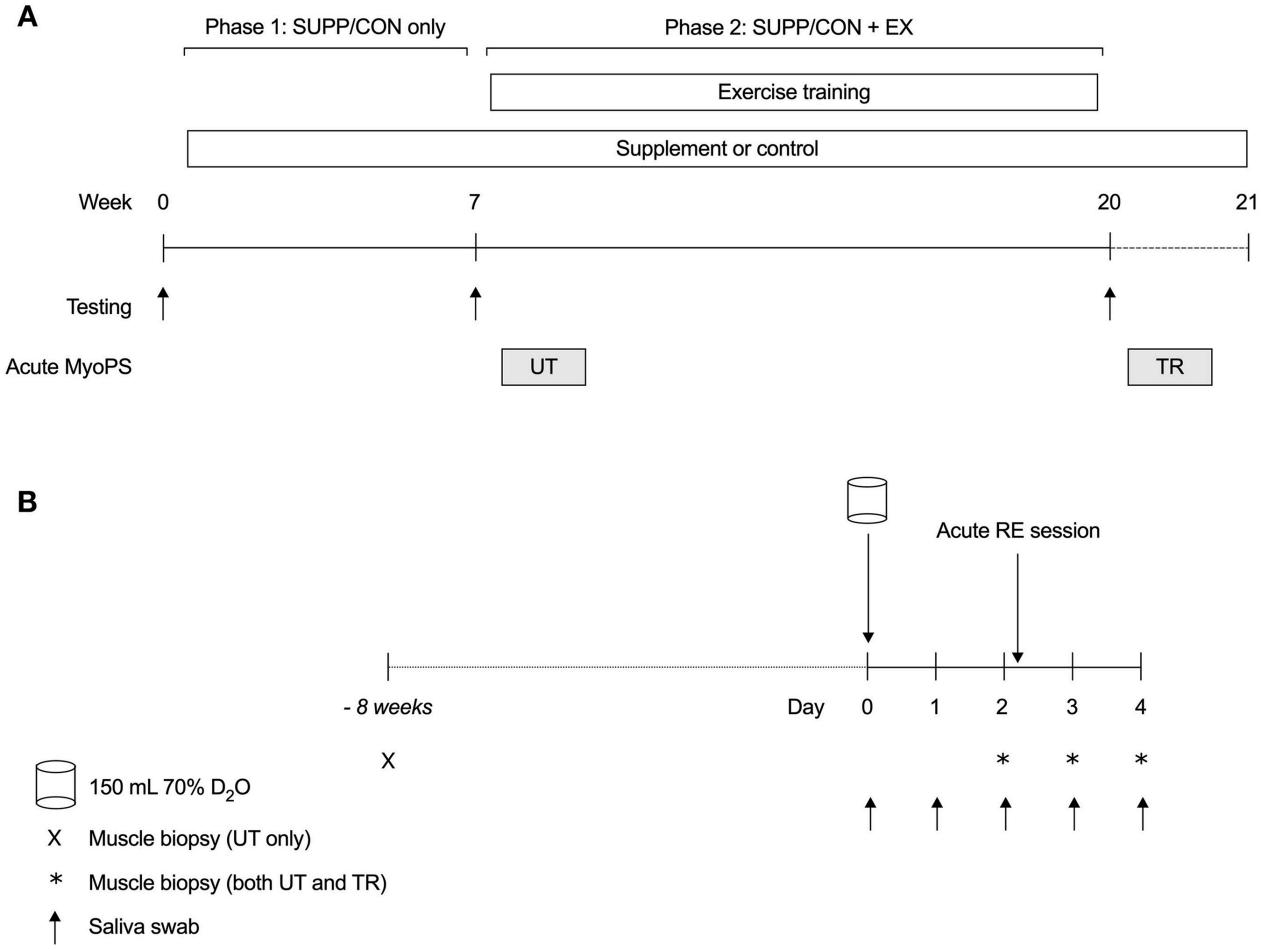
Overall study schematic **(A)** and acute MyoPS response protocol **(B)**. **(A)** Participants were randomly assigned to consume a multi-ingredient supplement (SUPP, *n* = 8) or control (CON, *n* = 7) beverage twice per day for 21 weeks. Between weeks 8 and 19, inclusive, participants completed a 12 weeks combined RET (twice per week) + HIIT (once per week) exercise training program. Lean tissue mass (DXA) and strength (1RM) were assessed at baseline (week 0), as well as pre- (week 7; Phase 1: SUPP/CON) and post-training (week 20; Phase 2: SUPP/CON + EX). The integrated MyoPS response to acute resistance exercise was assessed during participants' initial RET session (UT, untrained; week 8) and 10 days following their last RET session (TR, trained; week 21). **(B)** Following a baseline saliva sample, participants consumed 150 mL 70% deuterated water (D_2_O; Day 0). On Days 2–4, we obtained a fasting muscle sample from the *vastus lateralis*. Immediately after their muscle biopsy on Day 2, participants completed a session of resistance exercise at 65% 1RM. Saliva samples were collected regularly throughout each acute response period to assess deuterium (^2^H) enrichment of total body water. Eight weeks prior to the untrained acute response (–*8 weeks*), we obtained an unenriched, fasted muscle sample for the measurement of resting FSR. SUPP, supplement; CON, control; RET, resistance exercise training; HIIT, high-intensity interval training; DXA, dual-energy x-ray absorptiometry; 1RM, one repetition maximum; MyoPS, myofibrillar protein synthesis; ^2^H, deuterium; D_2_O, deuterated water; UT, untrained; TR, trained; FSR, fractional synthesis rate.

Following baseline strength, aerobic fitness, and body composition assessments, eligible and consenting participants were randomly assigned to consume an experimental nutritional supplement (SUPP *n* = 8; 30 g whey protein, 2.5 g creatine, 500 IU vitamin D; 400 mg calcium; 1,500 mg n-3 PUFA) or a carbohydrate-based control drink (CON *n* = 7; 22 g maltodextrin). Participants consumed their designated study beverages twice per day for 21 weeks (see Figure 1A). Strength and body composition were reassessed at weeks 7 (Phase 1: SUPP/CON only) and 20 (Phase 2: SUPP/CON + EX). From weeks 8 to 19 inclusive, participants completed a 12 weeks progressive exercise training program, which consisted of whole body RET twice weekly (Mondays and Fridays) and HIIT on a cycle ergometer once per week (Wednesdays).

The 0–24 h and 0–48 h integrated MyoPS response to acute resistance exercise was assessed during participants' initial RET session (UT, untrained; week 8; see Figure 1B), and 10 days following their final RET session (TR, trained; week 21). Participants continued to take the study beverages twice daily throughout weeks 8 and 21, including during the 48 h post-exercise recovery period.

### Training Outcomes

Whole body lean soft tissue mass (i.e., fat- and bone-free mass) and % body fat were measured by DXA (GE-LUNAR iDXA; Mississauga, ON). One-repetition maximum (1RM) strength tests were conducted for leg press, chest press, horizontal row, shoulder press, lateral pulldown, and leg extension. We assessed aerobic fitness using a peak oxygen uptake (VO_2_peak) test on a cycle ergometer. Particulars of these procedures can be found in our original trial (6). Type I and type II muscle fiber CSA were measured at weeks 0, 7, and 20 using immunohistochemistry. Muscle fiber CSA measurements were made on resting muscle samples only [i.e., −8 weeks (baseline), and Day 2 (0 h) during the UT and TR acute MyoPS assessments]. For details, please refer to our previous publications (14, 15).

### Tracer Protocol

Following collection of a baseline saliva sample (Day 0; Figure 1B) for the measurement of background deuterium (^2^H) enrichment of body water, participants consumed a single bolus dose of 150 mL 70% deuterated water (D_2_O). Serial saliva samples were obtained on Days 1–4 to capture the change in ^2^H enrichment of body water in response to D_2_O ingestion (Figure S1). Participants reported to the laboratory after an overnight fast the mornings of Days 2, 3, and 4 for a muscle biopsy (~30–50 mg) from the *vastus lateralis* muscle using a custom-modified 5 mm Bergstrom biopsy needle as described elsewhere (16). Biopsies were taken alternately from the left and right legs at least 5 cm apart beginning distally and moving proximally with successive biopsies. Directly following their biopsy on Day 2, participants completed a single session of resistance exercise.

A baseline muscle biopsy for the measurement of background ^2^H enrichment in skeletal muscle was obtained at the beginning of the study (8 weeks prior to the UT acute MyoPS response). Due to the already high number of biopsies per participant, we elected not to obtain a second “baseline” muscle biopsy immediately prior to the TR acute MyoPS response. As such, the measurement of resting (pre-acute RE) MyoPS was possible only in the UT state in this study.

### Acute Resistance Exercise

Each session began with a 5 min warm-up at 25 W on a cycle ergometer (ISO1000 Upright Bike; SCIFIT, Tulsa, OK). Participants then completed three sets of four exercises at 65% 1RM in the following order: leg press, chest press, horizontal row, and leg extension (HUR; Northbrook IL). The first two sets of each exercise consisted of 10–12 repetitions. The last set was performed to volitional fatigue, which we defined as the inability to smoothly move the weight through a full range of motion. Sets were separated by 1–2 min, and the workout was concluded with a 5 min cool-down on the cycle ergometer. In both the UT and TR states, 1RM was assessed 5–7 days prior to the acute resistance exercise session.

### Muscle Protein Synthesis

Body water ^2^H enrichment was measured as previously described (17). Briefly, 100 μL of saliva was placed in an inverted autosampler vial for 4 h at 100°C to extract body water. Vials were then immediately placed on ice in an upright position, and condensed body water was transferred to a clean autosampler vial. We then injected 0.1 μL body water into a high-temperature conversion elemental analyzer (Thermo Finnigan, Thermo Scientific, Hemel Hempstead, UK) connected to an isotope ratio mass spectrometer (Delta V Advantage, Thermo Scientific).

To measure ^2^H incorporation into myofibrillar proteins, we homogenized muscle samples (~30–50 mg) on ice and centrifuged them for 10 min at 2,300 g and 4°C to separate the myofibrillar and sarcoplasmic sub-fractions. The myofibrillar sub-fraction was purified, the protein-bound amino acids released by acid hydrolysis, and the sample eluted from an ion exchange resin as outlined elsewhere (16). Dried samples were then converted to their *n*-methoxycarbonyl methyl ester derivatives (18) for analysis by gas chromatography-*pyrolysis*-isotope ratio mass spectrometry (GC-*pyrolysis*-IRMS; Delta V Advantage, Thermo Scientific).

### Calculations

The fractional synthesis rate (FSR) of myofibrillar proteins was calculated using the standard precursor-product method (17):

$$\begin{array}{l} \text{FSR }\left(\text{\%}{\text{d}}^{-1}\right)=\left[\frac{E_{Ala2}-E_{Ala1}}{E_{BW}\times \;t}\right]\times \;3.7\;\times \;100 \end{array}$$

Where *E*_*AlaX*_ is the protein-bound enrichment (in atom percent excess) from muscle samples at time *X*. Therefore, the difference between time points is the change in protein-bound alanine enrichment between two time points with appropriate correction for ^2^H incorporation into alanine (17, 19). *E*_*BW*_ is the mean ^2^H enrichment (in atom percent excess) in total body water between time points. Two-day resting FSR was calculated using the difference in muscle protein ^2^H enrichments between Day 2 and baseline (collected at –*8 weeks*; Figure 1B); FSR at 0–24 h and 0–48 h post-resistance exercise were calculated using the difference between Days 2–3 and Days 2–4, respectively. Lastly, *t* is the tracer incorporation time in days. Multiplication by 3.7 adjusts for the average number of ^2^H atoms that are incorporated into alanine (17, 19), and multiplication by 100 converts the values to percentages.

### Statistical Analysis

Baseline physical characteristics between the two groups were compared using two-tailed Student's *t*-tests. The following outcomes were evaluated using two-way repeated measures ANOVA with group (SUPP or CON) and time as between- and within-subject factors, respectively, body composition and strength (0, 7, and 20 weeks); and FSR (rest, 0–24 h UT, 24–48 h UT 0–48 h UT, 0–24 h TR,24–48 h TR and 0–48 h TR). Muscle fiber CSA was evaluated using two-way repeated measures ANOVA with group (SUPP or CON) as a between-subjects factor, and time (0, 7, and 20 weeks) and fiber type (type I or type II) as within-subject factors. Notably, DXA, strength, and fiber size data for these subjects have been reported elsewhere (6, 14, 15) (albeit from different participant cohorts containing individuals not included in the tracer analysis), and are also presented in this study for the reader's convenience. Any significant *F* ratios were further scrutinized using Tukey's *post hoc* test. We examined the effect sizes of the changes in FSR using Cohen's D. Associations between FSR and changes in whole body and leg lean mass over training were examined using two-tailed Pearson correlations. For all analyses, statistical significance was accepted as *p* < 0.05. Data are presented in text and tables as mean ± SD.

## Results

### Participants and Compliance

At baseline, participants were 72 ± 7 years of age and overweight according to BMI (Table 1). No significant differences in age or baseline measures of lean tissue mass (whole body or regional), strength, or aerobic fitness were observed between groups. Compliance (assessed by questionnaire and returned drink sachets) with the nutritional supplements was 95 ± 4% (SUPP) and 95 ± 7% (CON). Participants attended 97 ± 3% (SUPP) and 94 ± 5% (CON) of their training sessions, and no participant missed more than two HIIT or RET sessions.

**Table 1:** Baseline characteristics of participants.

	**SUPP (*n* = 8)**	**CON (*n* = 7)**	***p*-value**
Age (years)	71 ± 7	73 ± 7	0.78
Weight (kg)	78.9 ± 11.2	83.0 ± 13.4	0.53
Height (m)	1.71 ± 0.06	1.75 ± 0.09	0.31
BMI (kg/m^2^)	26.9 ± 3.0	27.0 ± 3.4	0.96
% body fat	29.6 ± 6.5	30.6 ± 5.8	0.76
Whole body lean mass (kg)	53.1 ± 5.5	55.0 ± 7.2	0.57
Leg lean mass (kg)	18.4 ± 2.3	19.2 ± 3.3	0.61
VO_2_peak (mL/kg/min)	25.6 ± 4.3	25.7 ± 5.9	0.99
Leg extension 1RM (kg)	27 ± 6	28 ± 6	0.61
Leg press 1RM (kg)	80 ± 13	73 ± 27	0.54

*Values are means ± SD*.

### Exercise Training Adaptations

We observed a main effect of time for whole body lean mass (*p* < 0.01; Table 2), leg lean mass (*p* = 0.01), appendicular lean mass (*p* < 0.01), and % body fat (*p* = 0.02). No changes were observed during Phase 1, however over Phase 2 participants gained an average of 0.6 kg whole body (SUPP: +0.6 kg; CON: +0.5 kg), 0.3 kg leg (SUPP: +0.3 kg; CON: +0.3 kg), and 0.4 kg appendicular (SUPP: +0.3 kg; CON: +0.3 kg) lean mass, with no significant difference between groups. Percent body fat did not change during Phase 1, but decreased 0.9% after Phase 2 (SUPP: −0.8%; CON: −1.0%) with no difference between groups. We observed a group by time interaction for trunk lean mass (*p* = 0.015), such that the SUPP group gained 0.5 kg over Phase 1 with no further gains during exercise training in Phase 2. Trunk lean mass did not change over the course of the study in the CON group.

**Table 2:** Body composition and strength changes over training.

	**SUPP (*****n*** **=** **8)**			**CON (*****n*** **=** **7)**		
	**Baseline** **(week 0)**	**Pre-training** **(week 7)**	**Post-training** **(week 20)**	**Baseline** **(week 0)**	**Pre-training** **(week 7)**	**Post-training** **(week 20)**
Whole body lean mass (kg)^1^	53.1 ± 5.6^a^	53.9 ± 6.4^a^	54.5 ± 6.3^b^	55.0 ± 7.2^a^	55.0 ± 7.2^a^	55.5 ± 6.5^b^
Leg lean mass (kg)^2^	18.4 ± 2.3^a^	18.6 ± 2.7^a^	18.9 ± 2.6^b^	19.2 ± 3.3^a^	19.2 ± 3.4^a^	19.5 ± 3.0^b^
Appendicular lean mass (kg)^1^	24.6 ±2.9^a^	25.0 ± 3.4^a^	25.3 ± 3.2^b^	25.5 ± 4.1^a^	25.7 ± 4.4^a^	26.0 ± 3.8^b^
Trunk lean mass (kg)^3^	24.8 ± 2.8^a^	25.3 ± 3.2^b^	25.5 ± 3.2^b^	25.7 ± 3.1^a^	25.4 ± 2.8^a^	25.6 ± 2.7^a^
% body fat^2^	29.6 ± 6.5^a^	28.8 ± 6.7^a^	28.0 ± 6.3^b^	30.6 ± 5.8^a^	31.3 ± 5.8^a^	30.3 ± 6.2^b^
Σ upper body 1RM (kg)^1^	104 ± 14^a^	112 ± 12^b^	127 ± 12^c^	96 ± 20^a^	95 ± 20^b^	107 ± 22^c^
Σ lower body 1RM (kg)^1^	107 ± 16^a^	113 ± 17^b^	144 ± 19^c^	102 ± 31^a^	105 ± 29^b^	127 ± 35^c^

*Values are mean ± SD*.

1 *Main effect of time, p < 0.01*.

2 *Main effect of time, p < 0.05*.

3 *Group by time interaction, p = 0.015*.

*Dissimilar letters indicate differences over time within each treatment group*.

Muscle fiber CSA was not different between groups, and did not change over the course of the study (Table 3).

**Table 3:** Muscle fiber cross-sectional area.

	**SUPP (*****n*** **=** **8)**			**CON (*****n*** **=** **7)**		
	**Baseline** **(week 0)**	**Pre-training** **(week 7)**	**Post-training** **(week 20)**	**Baseline** **(week 0)**	**Pre-training** **(week 7)**	**Post-training** **(week 20)**
**MUSCLE FIBER SIZE (μm**^**2**^**)**						
Type I	6,907 ± 2,110	6,880 ± 1,090	6,883 ± 1,252	8,630 ± 1,737	6,765 ± 1,324	7,626 ± 1,565
Type II	6,409 ± 2,268	6,449 ± 1,214	6,676 ± 601	5,327 ± 1,349	4,924 ± 1,078	5,281 ± 1,003

*Values are mean ± SD*.

*No significant differences*.

We observed a main effect of time for the sum of upper and lower body 1RM (both *p* < 0.001). Upper body strength increased 4% during Phase 1 (SUPP: +8%; CON: 0%), and a further 13% following Phase 2 (SUPP: +13%; CON: +11%), with no differences between groups. Lower body strength increased 5% during Phase 1 (SUPP: +6%; CON: +3%), and a further 27% following Phase 2 (SUPP: +27%; CON: +22%), with no differences between groups.

### Acute Resistance Exercise Session

The volume lifted (weight [in kg] *x* repetitions/set *x* number of sets) during the acute resistance exercise session was greater after vs. before the 12 weeks training program (*p* < 0.01), but was not different between groups either pre- (SUPP: 3093 ± 484 kg; CON: 3370 ± 1521 kg) and post-training (SUPP: 4107 ± 938 kg; CON: 3911 ± 2136 kg).

### Myofibrillar Protein Synthesis

**Figure 2:**
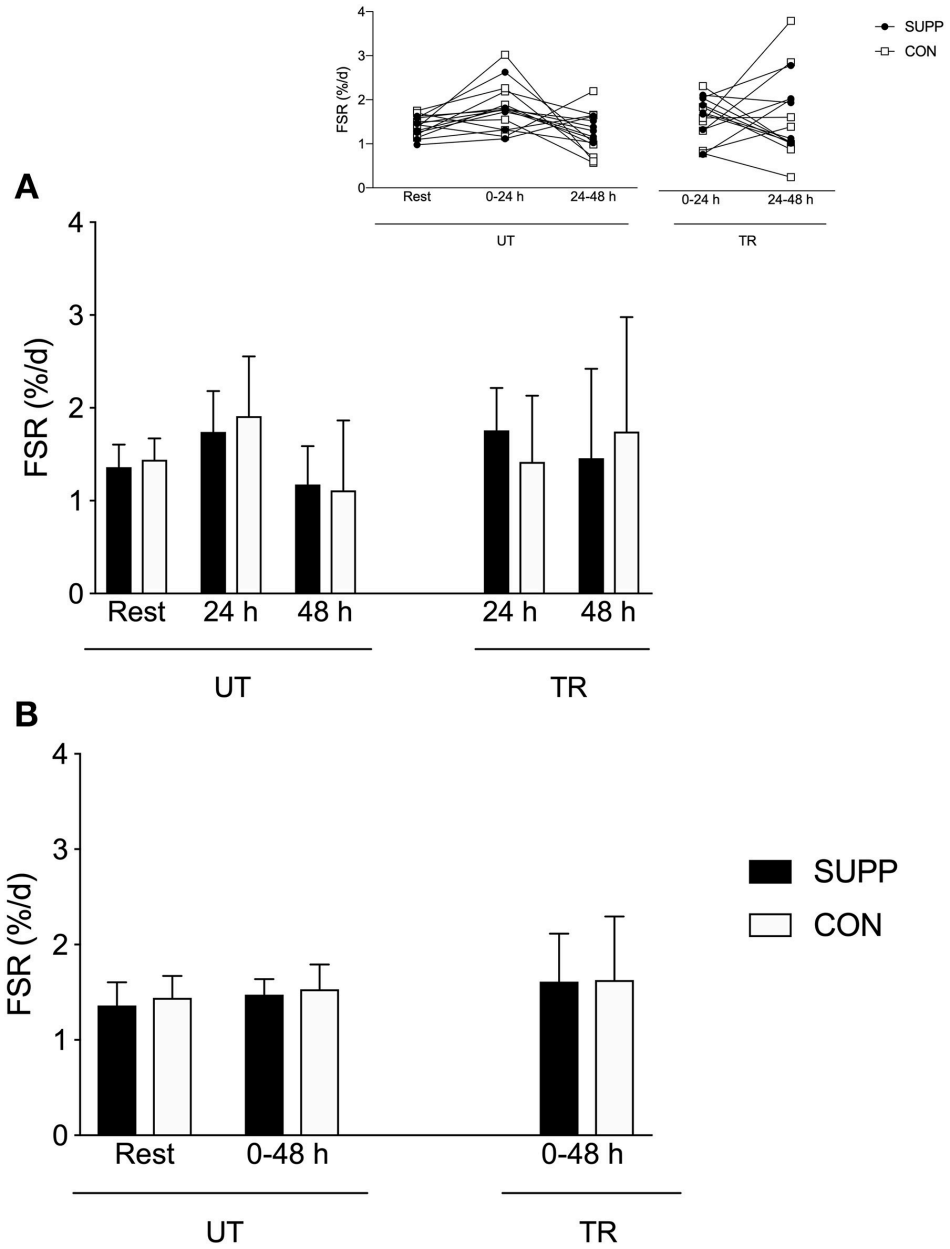
Integrated day-to-day **(A)** and cumulative **(B)** myofibrillar protein synthesis in response to acute resistance exercise pre- and post-training. Individual day-to-day (i.e., temporal) data are presented on the inset line graph in **(A)**. The SUPP group is presented in black; the CON group is presented in white. SUPP, supplement; CON, control; UT, untrained; TR, trained; FSR, fractional synthetic rate.

Resting FSR was similar in the SUPP (1.36 ± 0.24 %d^−1^; Figure 2) and CON (1.44 ± 0.23%d^−1^) groups. Although we observed trends for main effects of time for day-to-day (i.e., temporal; *p* = 0.08, Figure 2A) and cumulative FSR (*p* = 0.09, Figure 2B), these changes were not statistically significant. Following a bout of acute exercise in the untrained state, 0–24 h FSR was ~30% above resting rates (SUPP: 1.74 ± 0.44%d^−1^; CON: 1.91 ± 0.64%d^−1^) but 24–48 h FSR was slightly below rest (SUPP: 1.18 ± 0.41%d^−1^; CON: 1.11 ± 0.75%d^−1^; Figure 2A). However, when integrated over the entire 2-day post-exercise period (0–48 h), FSR was ~10% above resting rates (SUPP: 1.48 ± 0.16%d^−1^; CON: 1.53 ± 0.26%d^−1^; Figure 2B).

In the SUPP group post-training, 0–24 h FSR was ~30% above resting rates (1.76 ± 0.46%d^−1^, effect size: 0.86), and 24–48 h FSR was ~7% above resting rates (1.46 ± 0.96%d^−1^; Figure 2A); cumulative FSR over 0–48 h was ~20% above resting rates (1.61 ± 0.51%d^−1^; Figure 2B). In the CON group post-training, 0–24 h FSR was similar to resting rates (1.42 ± 0.71%d^−1^, effect size: 0.04), and 24–48 h FSR was ~20% above resting rates (1.75 ± 1.23%d^−1^; Figure 2A); cumulative FSR over 0–48 h was ~10% above resting rates (1.63 ± 0.67%d^−1^; Figure 2B). Again, whether expressed as day-to-day (*p* = 0.08) or cumulative (*p* = 0.09) values, FSR did not change significantly over time. We did not observe any between-group differences in FSR.

### Correlation Analysis

**Figure 3:**
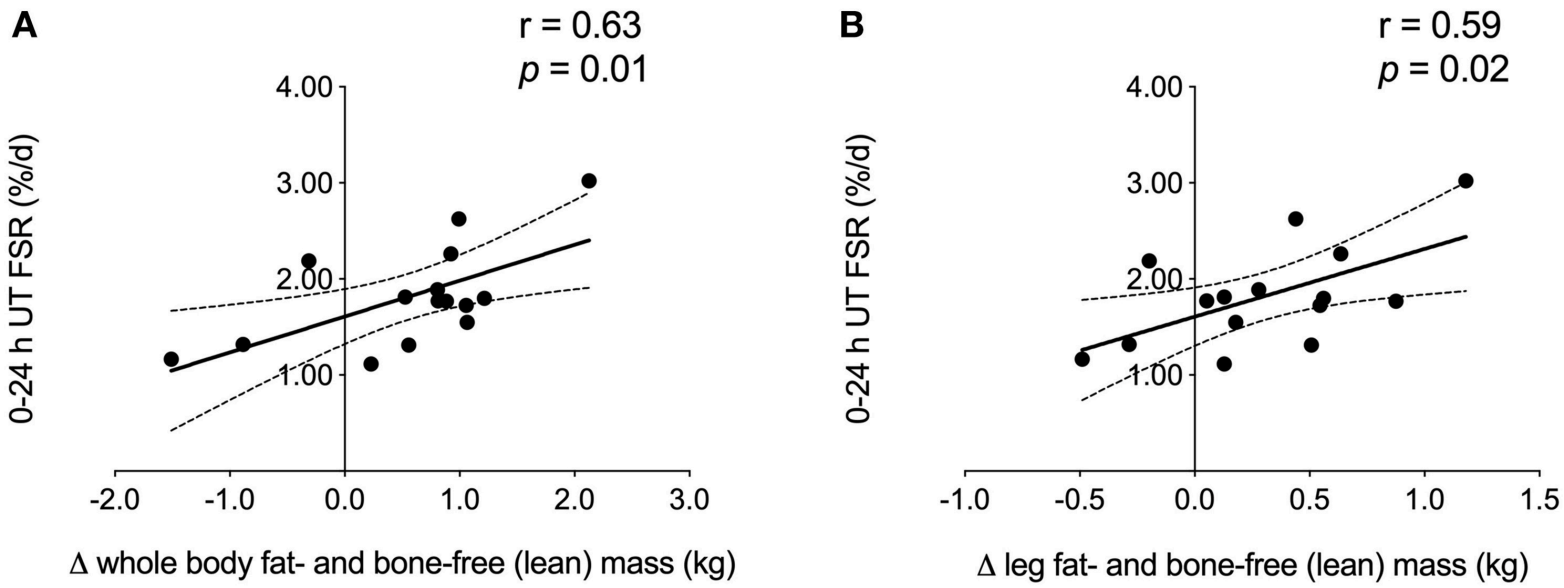
Correlation analysis. FSR 0–24 h after unaccustomed acute resistance exercise was positively associated with the change in **(A)** whole body and **(B)** leg fat- and bone-free (i.e., lean) mass during 12 weeks of multimodal (RET + HIIT) exercise training (Phase 2). Linear regression lines of best fit are shown in black. Dotted lines indicate 95% confidence intervals. UT, untrained; TR, trained; FSR, fractional synthesis rate; RET, resistance exercise training; HIIT, high-intensity interval training.

When collapsed across group, we observed a significant positive correlation between 0 and 24 h UT FSR and the amount of whole body and leg fat- and bone-free (i.e., lean) mass (Figure 3) gained over the course of exercise training. No other FSR time points were associated with changes in DXA measurements of lean body mass or muscle fiber CSA.

## Discussion

To our knowledge, this is the first study to examine the influence of a comprehensive multimodal exercise training program combined with a multi-ingredient nutrition intervention on integrated rates of MyoPS in previously inactive but healthy older men. We observed that 7 weeks of whey protein-based multi-ingredient supplementation did not augment the MyoPS response to an acute bout of RE. Furthermore, we were unable to detect a statistically significant increase in MyoPS to acute resistance exercise following the completion of a 12 weeks RET + HIIT program, despite sustained supplementation. We did, however, show a significant association between FSR 0 and 24 h following unaccustomed resistance exercise and lean tissue mass gains made over the course of the exercise training program, which is in contrast to our previous work showing no correlation between integrated post-exercise FSR assessed at the outset of a resistance training program and skeletal muscle hypertrophy (12).

The ingestion of whey protein independently stimulates MyoPS for 3–4 h (3, 20) as well as amplifies the rise in MyoPS immediately following resistance exercise in older men (3). Emerging evidence suggests that other ingredients such as vitamin D and n-3 PUFA may contribute to further increases in muscle protein synthesis (MPS) during hyperaminoacidemia (21–23). In middle-aged (24) and older (25) overweight and obese adults, 2–3 weeks of energy restriction has been shown to reduce both postabsorptive and postprandial rates of MyoPS, even when protein intake is maintained at 1.3 g/kg/d (i.e., above the recommended daily allowance [RDA] of 0.8 g/kg/d). In younger adults, 3 weeks of energy restriction depressed postprandial rates of mixed MPS despite protein intakes of 2–3 times the RDA (26). However, the impact of prolonged higher protein diets on resting postabsorptive and postprandial MyoPS in the absence of energy restriction is not well-described. To our knowledge, only two studies (27, 28) to date have measured resting MPS after *several weeks* of dietary intervention where weight loss was not an intended outcome. Hursel et al. (28) observed no difference in postabsorptive mixed MPS between young adults who underwent 12 weeks of a deficient (0.4 g/kg/d) or very high (2.4 g/kg/d) protein diet. Similarly, Gorissen et al. (27) observed no difference in postabsorptive or postprandial MyoPS between older men who completed 2 weeks of a lower (0.7 g/kg/d) or higher (1.5 g/kd/d) protein diet. The lack of change in postabsorptive and postprandial MPS following 2–12 weeks of increased protein intake in these two studies supports our observation in the present study that resting integrated rates of MyoPS (which incorporate both fasted and fed periods, as well as habitual physical activity) were unaffected by 7 weeks of whey protein-based multi-ingredient supplementation [which raised protein intake from 1.1 to 1.6 g/kg/d in the SUPP group (6)]. Considering the well-described anabolic effects of protein (3, 23), these findings are somewhat unexpected but may represent an adaptive response to longer-term increased protein intake.

Given that resistance exercise is a more potent anabolic stimulus compared to dietary protein (3, 29, 30), it is surprising that we did not observe an increase in integrated MyoPS 0–24 h or 0–48 h after acute resistance exercise in this previously sedentary group of older men. In the untrained state, rates of MyoPS were 38% (SUPP group) and 33% (CON group) higher than resting rates at 24 h post-resistance exercise, and had subsequently decreased in both groups by 48 h post-resistance exercise; yet, this trend for a change over time did not achieve statistical significance (*p* = 0.08 and *p* = 0.09 for temporal and cumulative FSR data, respectively). This is in contrast to our previous work showing substantial (~20–90%) increases in MyoPS 24 h post-resistance exercise in younger (12) and older adults (31); as well as more conservative (~15%), yet significant, increases in the 3-day integrated MyoPS response to acute resistance exercise in older adults (32). A key comparison can be made between the present study and Bell et al. (31), since both studies assessed integrated MyoPS 0–24 and 24–48 h following a similar bout of unaccustomed resistance exercise in older men. In Bell et al. (31) we reported a nearly 2-fold increase in MyoPS 0–24 h post-resistance exercise, which was slightly dampened (although still above resting rates) after 24–48 h. In contrast, we observed no significant increase in MyoPS at any timepoint following acute resistance exercise in the current study. A notable difference between the two studies is that resting (1.59 ± 0.03 vs. 1.40 ± 0.23%d^−1^) and 0–24 h post-exercise (3.10 ± 0.25 vs. 1.82 ± 0.53%d^−1^) FSR values were higher in Bell et al. (31) compared to this study, possibly due to differences in the D_2_O dosing protocol and/or time-frame over which the resting FSR measurements were made. In Bell et al. (31), participants ingested 120–180 mL D_2_O daily throughout the experiment, and resting measurements were made over the 24 h immediately prior to the resistance exercise bout. In the present study, participants ingested a single bolus 150 mL dose of D_2_O pre- and post-training, and resting measurements integrated the 8 weeks of the study prior to exercise training (Phase 1). In addition, the standard deviations of the FSR measurements appeared larger in the current study, suggesting that this pool of participants was more heterogeneous compared to Bell et al. (31), reducing our ability to detect changes over time or between groups. Other factors that may have contributed to the disparate findings between these two studies include the higher age (72 ± 7 vs. 67 ± 4 years), greater adiposity (% body fat: 30.0 ± 6.0 vs. 24.4 ± 4.9), and lower muscularity (whole body lean mass: 54.0 ± 6.3 vs. 61.4 ± 5.9 kg) at baseline of participants in the current study, relative to the subjects in Bell et al. (31). In fact, when appendicular lean mass is included as covariate in the two-way repeated measures ANOVA for FSR, we observe a main effect of time (*p* = 0.04) whereby—prior to exercise training—MyoPS 0–24 h post-resistance exercise is significantly elevated above both resting and 24–48 h rates, with no difference between groups. Clearly, additional work in carefully controlled studies is required to fully understand the integrated MyoPS response to unaccustomed resistance exercise in overweight older men.

The null findings in the present study are in line with a number of other studies that were unable to detect a significant increase in MPS in older adults despite subjects performing relatively strenuous exercise (3–12 sets of 8–12 repetitions at ≥ 65% 1RM) (33–35). Older individuals demonstrate a blunted rise in the MyoPS response to acute resistance exercise compared to their younger counterparts (2). This anabolic resistance to exercise combined with our use of integrated MyoPS measurements may have “diluted” the rise in MyoPS immediately following an acute bout of unaccustomed resistance exercise. Our participants were overweight, which is another factor that may have blunted the post-exercise rise in MyoPS. Recent work in young adults has shown that obesity may attenuate the ability of acute resistance exercise to increase fed-state MyoPS (36). Notably, however, the overweight status of the older adults in our previously published studies (31, 32) did not prevent us from detecting acute exercise-induced increases in integrated MyoPS. Moving forwards, researchers should be mindful that the evidence supporting the stimulatory effect of unaccustomed resistance exercise on MyoPS is equivocal in older adults, and future studies should endeavor to elucidate the underlying reasons for the discrepancy between studies.

Work using stable isotope infusions has shown that, in both younger and older untrained adults, the degree to which unaccustomed resistance exercise increases acute MyoPS over 4 h is not related to training-induced gains in muscle volume (37) or lean tissue mass (33). A novel finding of the current study is that *integrated* rates of MyoPS 0–24 h in response to unaccustomed resistance exercise were positively associated with the magnitude of lean mass gained during 12 weeks of RET + HIIT in older men, as measured by DXA. The association we observed herein at the outset of training is not as robust as the correlation between FSR and direct measures (e.g., muscle fiber CSA and ultrasound measures of *vastus lateralis* CSA) of skeletal muscle hypertrophy that we previously reported in younger men after 3–10 weeks of habituation to training and attenuation of the initial damage response to resistance exercise (12). Possible explanations for the discrepancy between our findings and those of Damas et al. (12) include the distinct study populations (overweight older men vs. normal weight young men), as well as the difference in baseline myofiber size. The relatively large pre-training fiber CSA in the present study [~6,000–7,000 μm^2^ vs. ~4,500 μm^2^ in Damas et al. (12)] may have inhibited our ability to detect hypertrophy following exercise training. The 0.6 kg increase in whole body lean tissue mass that we observed following exercise training is modest compared to other data (38); however, when examined individually most subjects (11 out of 15) demonstrated a change in lean mass of at least +0.5 kg. The magnitude of change in these subjects exceeds the error threshold for DXA-derived lean mass measurements of roughly ±0.5 kg (39), suggesting there was accretion of lean mass. Importantly, although the increase in lean tissue mass over Phase 1 in the SUPP group was restricted to the trunk (+0.5 kg), the majority of lean tissue gained in both groups over exercise training was in the limbs (+0.4 kg). These observations are consistent with recent work by Mitchell et al. (40) demonstrating a significant increase in whole body and trunk lean mass by DXA (both roughly +1.5 kg), but no change in appendicular lean mass, in older men who consumed a higher protein diet (1.6 g/kg/d) for 10 weeks. The gains in trunk lean mass observed in the present study and in Mitchell et al. (40) are likely due to hypertrophy of visceral non-muscle lean tissue (e.g., organs), which has been demonstrated following increased protein intake in animal studies (41, 42). Following the addition of 12 weeks RET + HIIT, we did not detect further increases in trunk lean mass, despite sustained protein-based supplementation in the SUPP group. Appendicular lean mass significantly increased across all subjects during exercise training, which is supportive of muscle growth because skeletal muscle comprises the majority of soft lean tissue in the limbs. Further, we have previously shown in a sample of CON subjects (which included *n* = 7 subjects from the current study) that muscle fiber CSA tended to increase over exercise training (*p* = 0.066) (15). We do not report a change in fiber size in the present study, despite the increase in lean tissue mass, likely due to the large variability inherent to studies with small samples sizes. Although the training-induced gains in whole body (SUPP: +0.6 kg; CON: +0.5 kg, see Table 2) and appendicular (SUPP: +0.3 kg; CON: +0.3 kg) lean mass were similar between groups, a single bout of accustomed resistance exercise (i.e., post-training) resulted in a 30% increase in MyoPS in the SUPP group relative to resting measurements of MyoPS made prior to training (effect size: 0.86; Figure 2). In contrast, in the CON group, a bout of accustomed resistance exercise did not appear to elevate MyoPS relative to rest (effect size: 0.04). Therefore, despite the fact that we report no significant changes in FSR in this study, we propose that multi-ingredient supplementation, together with RET + HIIT, has the potential to enhance exercise-induced increases in MyoPS but that we lacked the statistical power to detect such differences.

There are several limitations to the present study. Our relatively small sample size may have limited our statistical power and contributed to the null findings. Additionally, the subset of participants included in this study demonstrated divergent body composition changes compared to what we report in the main clinical trial. In the main study, lean tissue mass increased during Phase 1 for subjects in the SUPP group, with no further increase during Phase 2; whereas no changes in lean tissue mass were detected in the CON group. As such, we cannot generalize the findings of the present study to the rest of the subjects in the clinical trial. Lastly, although we report an association between gains in lean tissue mass over training and rates of post-exercise MyoPS prior to training, it is difficult to use DXA-derived lean mass as a surrogate for myofibrillar protein. As we have previously acknowledged, DXA measures fat-free mass (using hydration) rather than skeletal muscle directly. Further, factors that affect hydration, such as plasma volume and muscle water content, may change with protein supplementation and exercise training. Therefore, caution should be used when interpreting these data.

In conclusion, several weeks of whey protein-based multi-ingredient nutrition supplementation did not appear to enhance integrated MyoPS at rest or in recovery from acute RE in this group of healthy older men. However, integrated MyoPS measurements made 24 h post-resistance exercise in the untrained state were positively associated with hypertrophic gains made during a 12 weeks RET + HIIT program.

## Ethics Statement

This study was carried out in accordance with the recommendations of the Hamilton Integrated Research Ethics Board (HIREB) with written informed consent from all subjects. All subjects gave written informed consent in accordance with the Declaration of Helsinki. The protocol was approved by HIREB.

## Author Contributions

GP and SP acquired funding for this trial. KB, TS, GP, and SP conceived of and designed the study. KB, TS, and DK collected the data. KB, MB, KS, PA, and SP conducted the biochemical and statistical analysis. KB wrote the original draft, and all authors read and approved the final version of the manuscript.

### Conflict of Interest Statement

SP reports receipt of competitive grant support, travel expenses, and honoraria for speaking from the US National Dairy Council. The remaining authors declare that the research was conducted in the absence of any commercial or financial relationships that could be construed as a potential conflict of interest.

## Supplementary Material

Figure S1 Body water ^2^H enrichment in the untrained **(A)** and trained **(B)** state. Data were analyzed using a two-way repeated measures ANOVA with group (SUPP or CON) and time (Days 1–4) as factors. Dissimilar letters indicate significant differences over time. Both pre- and post-training, body water APE increased to approximately 0.20% and decreased significantly in a linear fashion each day thereafter (*p* < 0.0001). No differences between groups were observed. ^2^H, deuterium; APE, atom percent excess; SUPP, supplement; CON, control.
