# Corrigendum: Integrated Myofibrillar Protein Synthesis in Recovery From Unaccustomed and Accustomed Resistance Exercise With and Without Multi-ingredient Supplementation in Overweight Older Men

**DOI:** 10.3389/fnut.2020.611389

**Published:** 2020-10-30

**Authors:** Kirsten E. Bell, Matthew S. Brook, Tim Snijders, Dinesh Kumbhare, Gianni Parise, Ken Smith, Philip J. Atherton, Stuart M. Phillips

**Affiliations:** ^1^Department of Kinesiology, University of Waterloo, Waterloo, ON, Canada; ^2^School of Life Sciences, University of Nottingham, Nottingham, United Kingdom; ^3^Department of Human Biology, NUTRIM School of Nutrition and Translational Research in Metabolism, Maastricht University, Maastricht, Netherlands; ^4^Department of Medicine, University of Toronto, Toronto, ON, Canada; ^5^Exercise Metabolism Research Group, Department of Kinesiology, McMaster University, Hamilton, ON, Canada; ^6^School of Graduate Entry Medicine and Health, University of Nottingham, Derby, United Kingdom

**Keywords:** fractional synthesis rate, deuterated water, resistance exercise training, high-intensity interval training, whey protein, creatine, vitamin D, n-3 PUFA

In the original article, there was an error in Conflict of Interest Statement. The updated statement appears below.

The authors apologize for this error and state that this does not change the scientific conclusions of the article in any way. The original article has been updated.

## Conflict of Interest Statement

SP is listed as an inventor on patent (Canadian) 3052324 issued to Exerkine, and a patent (US) 16/182891 pending to Exerkine (but reports no financial gains). SP reports personal fees from Enhanced Recovery (donated to charity), equity from Exerkine (all proceeds donated to charity), outside the submitted work.

The remaining authors declare that the research was conducted in the absence of any commercial or financial relationships that could be construed as a potential conflict of interest.

