# Supplementary figures and images for: Integrated Myofibrillar Protein Synthesis in Recovery From Unaccustomed and Accustomed Resistance Exercise With and Without Multi-ingredient Supplementation in Overweight Older Men
Source: Front Nutr. 2019 Apr 11;6:40. doi: 10.3389/fnut.2019.00040 (PMC6470195; doi:10.3389/fnut.2019.00040)

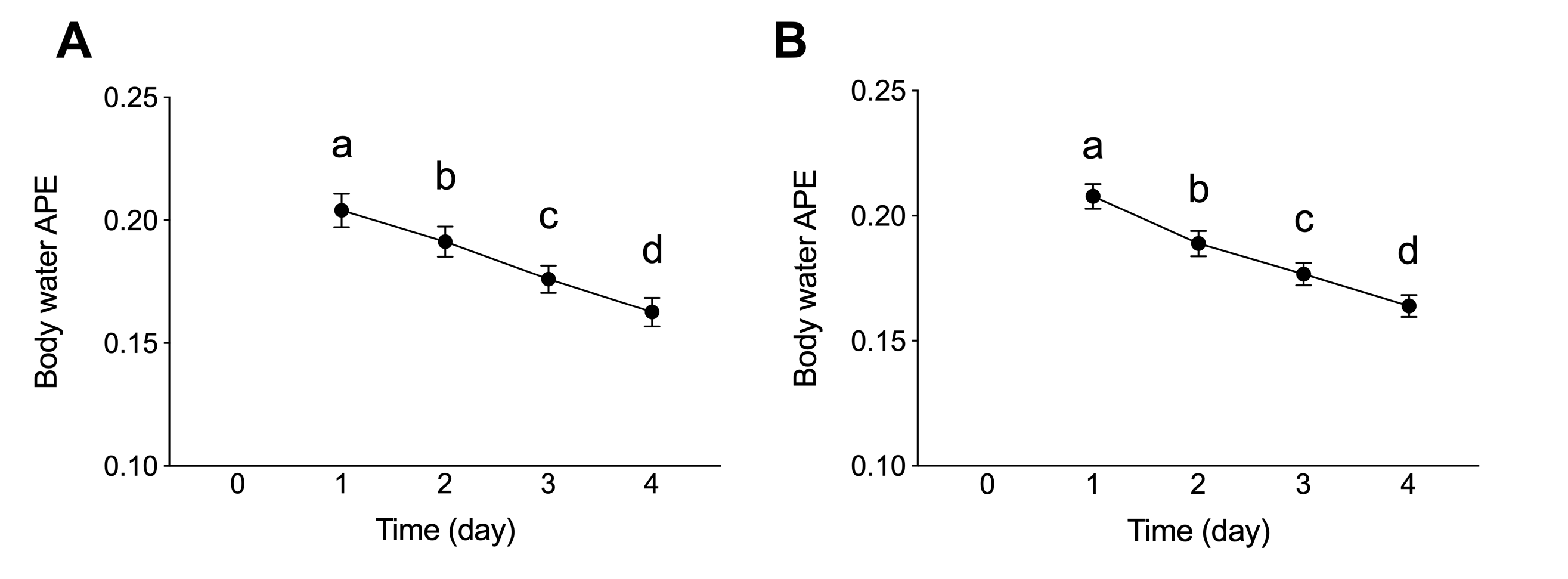

Supplement: Figure S1 — Body water 2H enrichment in the untrained (A) and trained (B) state. Data were analyzed using a two-way repeated measures ANOVA with group (SUPP or CON) and time (Days 1–4) as factors. Dissimilar letters indicate significant differences over time. Both pre- and post-training, body water APE increased to approximately 0.20% and decreased significantly in a linear fashion each day thereafter (p < 0.0001). No differences between groups were observed. 2H, deuterium; APE, atom percent excess; SUPP, supplement; CON, control. [file Image_1.TIFF]
